# Effects of Neck Taping in the Treatment of Hemispatial Neglect in Chronic Stroke Patients: A Pilot, Single Blind, Randomized Controlled Trial

**DOI:** 10.3390/medicina55040108

**Published:** 2019-04-17

**Authors:** Valentina Varalta, Daniele Munari, Lucrezia Pertile, Cristina Fonte, Gabriella Vallies, Elena Chemello, Marialuisa Gandolfi, Angela Modenese, Nicola Smania, Alessandro Picelli

**Affiliations:** 1 Neuromotor and Cognitive Rehabilitation Research Center, Department of Neurosciences, Biomedicine and Movement Sciences, University of Verona, 37134 Verona, Italy; valentina.varalta@univr.it (V.V.); lucrezia.pertile@gmail.com (L.P.); cristina.fonte@univr.it (C.F.); gabriella.vallies@gmail.com (G.V.); elena.chemello@univr.it (E.C.); marialuisa.gandolfi@univr.it (M.G.); angela.modenese85@gmail.com (A.M.); alessandro.picelli@univr.it (A.P.); 2 Neurorehabilitation Unit, Department of Neurosciences, Hospital Trust of Verona, 37126 Verona, Italy; daniele.munari@aovr.veneto.it

**Keywords:** athletic tape, perceptual disorders, rehabilitation

## Abstract

*Background and objectives:* Hemispatial neglect is a common consequence of stroke, with an estimated incidence of 23%. Interventions for treating hemispatial neglect may be categorized as either top-down or bottom-up processing. The aim of top-down approaches is to train the person to voluntarily compensate for their neglect. Such approaches require awareness of the disorder and a high level of active participation by the patient. Differently, bottom-up approaches are based on manipulation of a patient’s sensory environment and so require less awareness of behavioral bias. In line with the latter, it is conceivable that elastic therapeutic taping applied to the left neck surface may provide bottom-up inputs that reduce hemispatial neglect symptoms. The aim of this study was to assess the effect of therapeutic neck taping on visuo-spatial abilities, neck motion, and kinesthetic sensibility in chronic stroke patients with hemispatial neglect. *Materials and Methods:* After randomization, 12 chronic stroke patients with hemispatial neglect received 30 consecutive days of real (treatment group) or sham (control group) neck taping. The outcomes were as follows: Stars Cancellation Test; neck active range of motion; Letter Cancellation Test; Comb and Razor Test; Cervical Joint Position Error Test evaluated before and after one month of taping. *Results:* Between-group comparison showed significant differences only for the Cervical Joint Position Error Test after treatment (*p* = 0.009). *Conclusions:* Our preliminary findings support the hypothesis that neck taping might improve cervicocephalic kinesthetic sensibility in chronic stroke patients with hemispatial neglect. Further studies are needed to strengthen our results and better investigate the effects of elastic therapeutic taping on visuo-spatial abilities in stroke patients with hemispatial neglect.

## 1. Introduction

Stroke is a leading cause of disability in adults [1]. Its sequelae may affect both sensorimotor systems and cognitive functions [2,3]. Hemispatial neglect is a common consequence of stroke, with an estimated incidence of 23% (range 8 to 95% in stroke patients) [4,5]. Cortical and subcortical stroke lesions involving the right hemisphere are frequently associated with hemispatial neglect [6,7]. Patients suffering from hemispatial neglect have been noted to fail to report, respond to, or be aware of stimuli located contralateral to the brain lesion [8,9]. From a functional point of view, the presence of hemispatial neglect may increase postural control abnormalities after stroke, leading to trunk misalignment [10], postural instability [11,12], and increased risk of falls [13,14,15].

Hemispatial neglect rehabilitation is focused on inducing patients to explore their neglected space (usually left side). Sensory manipulation (vestibular, visual or somatosensory stimulation) has been proposed as treatment to compensate the distortion in processing spatial information. Such interventions may be categorized as bottom-up processing based on manipulation of a patient’s sensory environment [3]. External sensory stimulation (bottom) may passively activate the neglected side of the body and enhance hemisphere activation (up). Rehabilitation studies have suggested that stimulation needs to be repeated over time in order to obtain long-term improvement in hemispatial neglect. Bottom-up processing requires less awareness of the behavioral bias than top-down approaches, which, instead, require voluntarily compensating for the neglect and a high level of active participation by the patient [16,17,18,19,20,21]. One of the sensory manipulations for treating hemispatial neglect rehabilitation is transcutaneous electric nerve stimulation (TENS) [12,22,23]. The technique entails stimulating superficial cutaneous nerve Ia fibers using an electric current at an intensity below the motor threshold. When applied to the left neck muscles, TENS may activate the neglected side of the body, reducing hemispatial neglect [22,24] and associated signs such as balance disorders [12].

Since the 1970s, elastic therapeutic taping has become a very common treatment for a variety of conditions [25]. Among the diverse taping techniques, elastic therapeutic taping is capable of stretching up to 130–140% of its resting static length while ensuring free mobility and recruitment of the applied muscle or joint. After the tape is applied, the taped area forms convolutions, thus increasing the space between skin and muscles. Lifting the skin enhances the flow of blood and lymphatic fluid to the body area [26]. Like TENS, elastic taping may increase cutaneous stimuli and enhance somatosensory inputs [27]. The application of therapeutic tape under tension has been shown to change sensory feedback transmission [28,29]. Like TENS, taping may increase attention toward the left side by manipulating a patient’s sensory environment, and thus reduce hemispatial neglect. In this way, elastic therapeutic taping may act as a bottom-up approach. It is conceivable, therefore, that elastic therapeutic taping applied to the left neck surface might provide bottom-up inputs that reduce hemispatial neglect symptoms. Unlike TENS, elastic taping can continually activate the neglected side because kept applied for several consecutive days. Furthermore, taping is easier and cheaper than TENS.

To the best of our knowledge, no previous study to date has investigated the potential effect of elastic taping in patients with hemispatial neglect. The primary aim of this study was to assess the effect of therapeutic neck taping on visuo-spatial abilities in chronic stroke patients with hemispatial neglect. The secondary aim was to assess the effects of taping on neck motion and kinesthetic sensibility in such patients. In order to examine the possible rehabilitation power of this technique, we assessed patients after four weeks of tape application.

## 2. Materials and Methods

This was a single-center, pilot, randomized controlled trial (RCT). Inclusion criteria were as follows: age > 18 years; presence of ischemic or hemorrhagic stroke (as documented by computerized tomography or magnetic resonance imaging scan) that had occurred at least 6 months earlier; presence of hemispatial neglect (Star Cancellation Test score < 50) [30]; ability to actively rotate the head toward the left in a closed-eyes condition (absence of musculoskeletal disorders). Exclusion criteria were: participation in other trials, dementia (Mini Mental State Examination correct score < 23.80 [31]; presence of severe of comprehension deficits, psychiatric disorders, deficits of somatic sensation involving the cervical dermatome map (C3–C5), visual field deficits as assessed by neurological examination; other neurological or orthopedic conditions involving the neck, and visual ability.

All participants were outpatients. Written, informed consent before participation in the study was obtained from all patients. The study was carried out according to the Declaration of Helsinki and approved by the local Ethics Committee (Clinical Trials registration number NCT03263455).

### 2.1. Treatment Procedures

Prior to testing, eligible participants were allocated to one of two study arms according to a balanced (restricted) randomization scheme: treatment group (TG) group and control group (CG).

All patients were evaluated by the same investigator who was blinded to group allocation. In order to prevent selection bias, we used an allocation concealment mechanism (sequentially sealed numbered containers). The success of blinding was tested by asking the assessor to make an educated guess. Another investigator checked for correct patient allocation according to the randomization list. After unmasking at the end of the study, we checked that no allocation errors had been made.

All participants were taped by the same investigator who was not involved in the outcome assessment. A standard elastic therapeutic tape was used for all applications. I-strips were applied, as they have proven effective in treating pain symptoms [26]. The TG received a standardized therapeutic application of elastic tape (real taping), while the CG received sham taping. The intervention entailed wearing the elastic tape for a total of 30 days. The tape was replaced every 4 days. The participants performed no rehabilitation other than what was scheduled in the study protocol.

**Figure 1:**
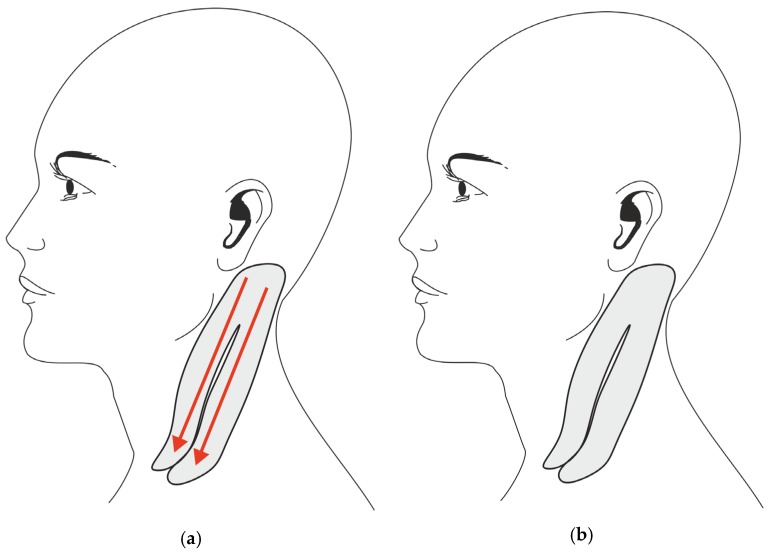
Taping application. (**a**) Taping application in the Treatment Group (red arrows indicate the direction of tape tension). (**b**) Sham taping in the Control Group (no tape tension).

For the TG, the tape was placed according to Kenzo Kase's KinesioTaping Method [26] by an experienced physiotherapist. The tape strip was applied from the mastoid bone to the clavicle (rostrocaudal direction) with the sternocleidomastoid kept in a position of maximum stretching. Two I-strips were applied: the first placed on the medial (sternal) head and the second on the lateral (clavicular) head of the sternocleidomastoid muscle with 15% to 25% of tension (paper-off tension) (Figure 1a).

For the CG, smaller I-strips were used. To eliminate the specific therapeutic elements of elastic taping (i.e., longitudinal stretch, start, and ending points of tape application), the strips were applied without tension and without stretching the muscles, perpendicularly to the muscle belly (starting from the middle and progressing to each side) over the same neck muscles as in the TG (see Figure 1b).

### 2.2. Testing Procedures

During the study period, the subjects underwent cognitive, kinesthetic sensibility, and motor assessments before (T0) and after (T1) wearing the tape for 1 month. Specifically, the T0 assessment was conducted the day of the first application, whereas the T1 assessment was conducted the day after having removed the tape. The tape was temporarily removed for collecting measurements. The same raters evaluated all subjects.

The primary outcomes were changes in scores on the Stars Cancellation Test (SCT) and in degree of Active Range of Motion (AROM) of neck-left rotation. The SCT evaluates visuo-spatial attention and requires that the subject bars 56 targets (the small stars) embedded within 75 distractors (bigger stars, words and letters) on an A4 horizontal sheet of paper. The total score is the sum of all the targets (minus two targets in the center of the sheet) and the range is from 0 (worst performance) to 54 (best performance) [30]. To assess the patient’s ability to rotate the neck to the left, we measured the AROM in degrees by means of a goniometer [32], wherein the greater the ability to rotate the neck the more the degrees. Patients were seated during assessment.

The secondary outcomes were changes in the scores on the Letter Cancellation Test (LCT), Comb and Razor Test (CRT), AROM of the neck (right rotation, inclination, flexion at both sides, flexion and extension), and Cervical Joint Position Error Test (CJPET). The LCT evaluates visuo-spatial attention and requires that the subject bars 40 targets (the letters E and R) embedded within 130 different letters (distractors) divided in 5 lines on an A4 horizontal sheet of paper. The total score is the sum of all targets, ranging from 0 (worst performance) to 40 (best performance) [30].

The CRT is a functional assessment for hemispatial neglect patients that are required to comb their hair and then shave themselves (men) or put on make-up powder (women). The number of strokes (right, central, left) within 30 s is recorded. The percent bias between left and right stroke is calculated (the total number of strokes on the left side minus the total number of strokes on the right side divided by the total number of strokes for both sides plus any ambiguous strokes). Scores ranged between −1 (left neglect) to +1 (right neglect) [33].

To assess the patient’s ability to rotate-right, incline, flex, and extend the neck we measured AROM in degrees by means of a goniometer [32], wherein the greater the ability to move the neck the more the degrees. Patients were seated during assessment.

The CJPET assesses cervicocephalic kinesthetic sensibility. The test requires blindfolded subjects to relocate the head on the trunk, to a subjective straight-ahead position, after a near-maximal active movement of the head in the horizontal or vertical plane. A laser pointer was fixed on the head by a helmet aimed at a target 90 cm in front of the subject. A circle target holder was constructed (40 cm in diameter). The degrees between the center of the circle (0°) and the laser light position were recorded. Deviation of more than 4.5° indicates a deficit in proprioception [34].

### 2.3. Statistical Analysis

Statistical analysis was carried out using the Statistical Package for the Social Sciences (SPSS) software, version 20.0, for Macintosh (SPSS Inc., Armonk, NY, USA). We assessed all patients who were randomized (intention-to-treat principle). Normality of main outcomes was examined by means of the Kolmogorov-Smirnov test, which showed a normal distribution for the SCT (*p* = 0.454) and the AROM of neck-left rotation (*p* = 0.410). We used the unpaired *t*-test to assess sample homogeneity before treatment and to compare the effects of treatment between groups. To determine this, we computed the differences in performance at T1 and T0 for all outcomes. Within-group comparisons were performed using the paired *t*-test. Descriptive analysis was used to evaluate the effect size (*r*) measures between groups (Cohen’s d calculation) and the 95% confidence intervals (CI) [35]. The alpha level for significance was set at *p* < 0.05.

## 3. Results

Twelve subjects (6 males, 6 females; mean age 66.1 years) with hemispatial neglect (mean SCT score 37.0 ± 14.7) consequent to stroke (mean time since onset 14.6 months) were recruited from among 82 outpatients consecutively admitted to our Research Center between June and December 2015. Seventy patients were excluded from the study: 39 didn’t meet the inclusion criteria, 23 were enrolled in other trials, and 8 declined participation.

Table 1 presents the demographical and clinical features of the sample.

**Figure 2:**
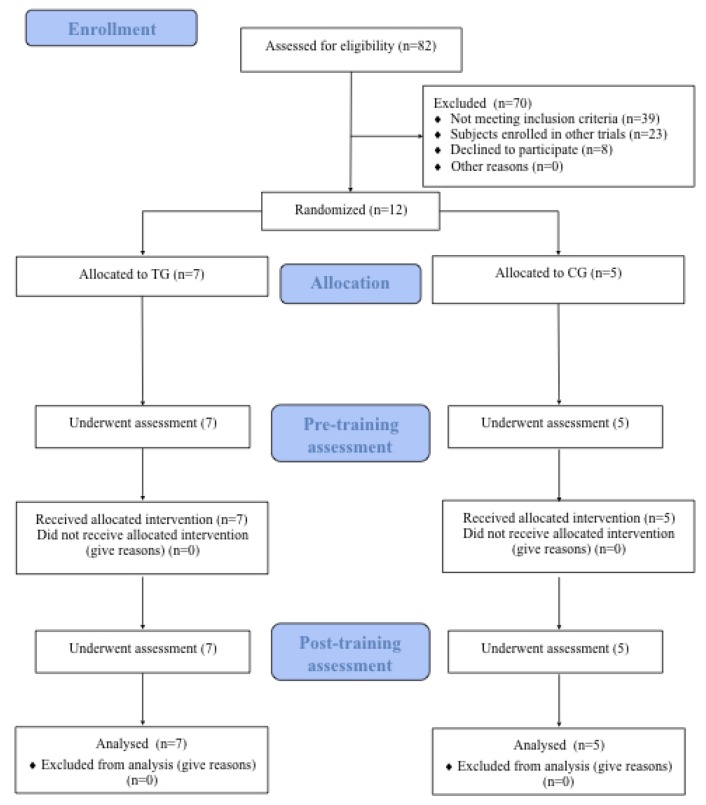
Study Flow.

No drop-outs or adverse events were recorded for either group during the study. The study flow diagram is illustrated in Figure 2.

As to the primary outcomes, a between-group comparison showed no significant difference in the SCT score and AROM of neck-left rotation (Table 2) between the TG and the CG. Within-group comparison in the TG showed significant changes in AROM at post-treatment assessment of neck-left rotation. No significant differences were noted in the CG (Table 3).

For the secondary outcomes, a between-group comparison showed significant differences in the AROM of neck-right rotation and the CJPET scores between the TG and the CG (Table 2). A within-group comparison showed that changes in performance of the TG were significant for post-treatment AROM of the neck (right rotation, inclination, flexion at both sides, and flexion), whereas no significant changes were found in the CG (Table 3).

## 4. Discussion

We conducted this pilot RCT to evaluate the effects of elastic therapeutic taping on hemispatial neglect in chronic stroke patients. Also, we tested the effects of taping on motor abilities and kinesthetic sensibility of the neck. Although no significant difference in effect on visuo-spatial abilities or on motor abilities was found between the two groups, neck AROM (particularly rotation and inclination in both sides and flexion) was improved in the TG patients after 4 weeks of application.

These results are shared by previous studies that suggested that elastic therapeutic taping may induce changes in sensory discrimination, with improved sensory input, decreased spasticity through proprioception feedback, and relief of abnormal muscle tension [26,27,36,37,38]. For instance, a study by Cho et al. investigating the effects of taping on pain, AROM, and proprioception in patients with knee osteoarthritis showed decreased pain with movement, increased joint range of motion as a result of increased muscle activation and improved proprioception [38]. Furthermore, a study by Alexander and coworkers provides convincing evidence for a change in sensory feedback transmission with the taping under tension in a manner similar to our study [28].

In our study, unlike the sham condition, the tape was applied by stretching the sternocleidomastoid muscle, thus creating a therapeutic effect in which tape forms wrinkles or convolutions in the skin. This wrinkling effect lifts the skin and increases the space between the muscle and the skin, promoting blood flow and lymph flow to the neck area and stimulating proprioception input [26,38].

Furthermore, the TG patients showed that they were better able to relocate the head on the trunk after active movement in the horizontal plane (CJPET), whereas the CG did not.

Consistent with our results, it is conceivable that taping might also induce a better representation of the neck in space and improve internal representation of the body in space, which is often compromised in stroke patients [12,39]. A study by Perennou et al. showed, in fact, that patients with hemispatial neglect displayed pronounced postural instability as compared with other patients without neglect and healthy controls. However, they found that TENS systematically increased postural control, so it could unmask their latent postural capacities [12]. From this point of view, the effects of elastic therapeutic taping on postural capacity seem to be similar to those of TENS.

We speculated that a possible explanation for the positive results seen after the application of elastic therapeutic taping could be a corrective proprioceptive input signal that changes subjective body orientation by modifying head-on-trunk representation [40,41] in the TG group patients. This could be due to the mechanical effects of taping: skin receptor output is increased, supraspinal centers are stimulated, and muscle activity adjusted via proprioception feedback [42]. These effects enhance kinesthetic and joint position sense [43,44], improve neck sensation, and increase muscle recruitment by reducing joint movement and increasing the overlap of muscular filaments [45]. Indeed, applying pressure to and stretching the skin with taping can (a) stimulate cutaneous mechanoreceptors which may contribute to improvement in dynamic activities and (b) enhance signal information of joint movement or joint position [46,47,48].

Hence, application of elastic taping might have increased the subjects’ proprioceptive senses and stability, as well as stimulated diverse sensory receptors, improving contraction ability of the muscles [49]. A previous study reported that elastic taping applied to subjects with ankle sprain enhanced their muscle strength and gait ability [50,51].

In the final analysis, we observed little improvement in visuo-spatial abilities after elastic therapeutic taping; the preliminary results show that these effects are not statistically significant. Nonetheless, to our knowledge, no study to date has addressed the use of elastic therapeutic taping for treating hemispatial neglect in stroke patients. Since therapeutic taping induces effects similar to TENS, it is possible to compare them. Indeed, both are bottom-up techniques because they induce passive activation of the neglected body side, thus potentially compensating for the rightward bias of hemispatial neglect. As compared with top-down techniques, treatments based on bottom-up mechanisms are potentially more successful because they pose fewer prerequisites for the functional status of patients with neglect: they do not necessarily require patient cooperation in attending and exploring the left side of space.

Our results seem to contrast those of Vallar et al. [24] who suggested that as long as patients receive stimulation to the left side of the body, hemispatial neglect could be positively affected. Differently, we found that hemispatial neglect was not significantly affected by either elastic therapeutic taping or sham taping.

Furthermore, previous studies reported positive effects of TENS on visuo-explorative tasks [22,23]. For instance, Guariglia et al. [22] showed that TENS to the left neck muscles significantly improved performance in the condition in which subjects identified the location of an object using only the environmental frame of reference, but not in the condition in which they had to integrate it with a relevant visual cue.

From this perspective, elastic therapeutic taping does not seem as efficient as TENS in reducing visuo-spatial deficits. To our knowledge, no previous study has investigated the effect of taping in hemispatial neglect patients; therefore, we can only assume the reasons why this is so. First, the patient sample was small. Second, the sensory stimulation provided by elastic therapeutic taping might differ from that by TENS. Specifically, the level of stimulation obtained with taping may not be enough to improve attention toward the neglected side after 4 weeks of application. Third, these patients were in a stage of chronic illness in which visuo-spatial deficits are generally stabilized and more difficult to modify than in acute and sub-acute stages. This third point is in line with evidence that recovery is faster and greater in the early stages after stroke than in the chronic phases; in other words, in the acute stage the brain is “primed” for recovery [52], whereas delays may limit the patient’s ability to recover.

Nevertheless, our results are interesting because they may produce potentially useful ideas other studies could use. Although we observed no significant effects of elastic therapeutic taping on visuo-spatial abilities, our findings suggest that taping could be a useful technique for improving motor abilities and kinesthetic sensibility of the neck. As compared with other bottom-up techniques (e.g., TENS), it is less expensive, can provide continuous stimulation, and can be easily applied even at home.

This study has several limitations. The sample size was small. We estimated that a total of 40 subjects (20 per group) would provide a power of 80% to detect a between-group difference of 10 points (standard deviation of 14.95 points) on the SCT. Second, no follow-up was conducted. Third, we did not test the global functional outcome.

These limitations notwithstanding, our findings indicate that a future area of focus is to combine taping with cognitive rehabilitation and to compare their combined effects with those of other techniques in order to better understand the potential effects of taping in neurorehabilitation. In the future, studies might be interested in investigating the effects of taping on hemispatial neglect applied during conventional treatment (motor or cognitive training) in order to enhance the effect of the training. Second, it may be useful to understand the effects of the two different taping application methods proposed by Kase [26] on visuo-spatial ability or kinesthetic sensibility. Third, a longer application of the taping could further increase the clinical effects. Finally, a follow-up assessment session at 3 to 6 months is required.

## 5. Conclusions

The preliminary results of this pilot study show greater improvement in cervicocephalic proprioception and neck reposition sense as assessed by CJPET after therapeutic elastic taping, as compared with sham taping in chronic stroke patients with hemispatial neglect. Taping had no effect on visuo-spatial abilities. Further studies are needed to strengthen our results and better investigate the effects of elastic therapeutic taping on visuo-spatial abilities in stroke patients with hemispatial neglect.

## Figures and Tables

**Table 1:** Demographical and clinical features of the sample.

	TG Group	CG Group	*p*-Value
Gender male/female	2/5	4/1	
Stroke ischemic/hemorrhagic	5/2	2/3	
Age (years) mean (SD)	65.5 ± 10.2	67.0 (11.5)	0.830
Education (years) mean (SD)	9.0 (3.4)	7.8 (3.9)	0.599
Time from onset (months) mean (SD)	19.7 (27.7)	28.0 (40.6)	0.908
BI modified (0–100) mean (SD)	77.4 (34.3)	57.0 (15.5)	0.247
MMSE (0–30) mean (SD)	26.4 (2.1)	25.4 (1.9)	0.406
Star Cancellation Test (0–50) mean (SD)	39.1 (14.9)	34.0 (5.4)	0.575
AROM Left Rotation (degrees) mean (SD)	14.5 (2.3)	17.2 (4.1)	0.176

Abbreviations: TG: Treatment Group; CG: Control Group; BI: Barthel Index; MMSE: Mini Mental State Examination; AROM: Active Range of Motion.

**Table 2:** Between-group comparisons.

Outcome Measures	TG vs. CG Comparison	*p*-Value	Effect Size (*r*)
Star Cancellation Test (0–50)		0.387	‒0.29
Letters Cancellation Test (0–40)		0.379	‒0.25
Comb and Razor Test (%)		0.772	‒0.10
AROM (degrees)	Rotation right	0.049 *	‒0.56
	Rotation left	0.432	‒0.21
	Inclination right	0.330	0.29
	Inclination left	0.499	0.20
	Flexion	0.107	‒0.47
	Extension	0.548	0.19
CJPET (degrees)		0.025 *	‒0.73

Abbreviations: TG: Treatment Group; CG: Control Group; AROM: Active Range of Motion; CJPET: Cervical Joint Position Error Test; * = Significant comparison (*p* < 0.05).

**Table 3:** Within-group comparisons.

Outcome	Group	T0	T1	Within-Group Comparisons
		Mean (SD)	Mean (SD)	*p*-Value (95% CI)
Star Cancellation Test	TG	39.14 (14.98)	41.71 (13.44)	0.203 (‒6.970; 1.827)
	CG	34 (15.42)	43.2 (3.27)	0.242 (‒27.813; 9.413)
Letters Cancellation Test	TG	26.29 (11.31)	29.14 (12.13)	0.118 (‒6.686; 0.972)
	CG	23.60 (11.61)	29.40 (10.00)	0.137 (‒14.465; 2.865)
Comb and Razor Test	TG	‒0.17 (0.46)	‒0.10 (0.13)	0.604 (‒0.618; 0.401)
	CG	‒0.31 (0.33)	‒0.22 (0.14)	0.196 (‒0.505; 0.159)
Neck AROM	Rotation right			
	TG	14.07 (3.27)	11.79 (2.67)	0.011 (0.732; 3.839) *
	CG	13.80 (3.27)	13.60 (3.11)	0.772 (‒1.589; 1.989)
	Rotation left			
	TG	14.50 (2.36)	12.64 (2.62)	0.012 (0.585; 3.130) *
	CG	17.20 (4.08)	17.20 (4.62)	1.000 (‒7.306; 7.306)
	Inclination right			
	TG	15.29 (2.50)	17.21 (2.23)	0.032 (‒3.626; ‒0.231) *
	CG	19.20 (2.59)	20.10 (2.30)	0.255 (‒2.783; 0.983)
	Inclination left			
	TG	14.71 (2.69)	17.57 (2.30)	0.025 (‒5.211; ‒0.504) *
	CG	18.30 (1.72)	20.10 (3.88)	0.198 (‒5.044; 1.444)
	Flexion			
	TG	2.34 (0.94)	1.57 (0.79)	0.028 (0.118; 1.425) *
	CG	1.60 (0.90)	1.50 (0.50)	0.704 (‒0.580; 0.780)
	Extension			
	TG	12.36 (1.4)	13.36 (2.36)	0.212 (‒2.751; 0.751)
	CG	14.90 (2.35)	15.30 (2.11)	0.495 (‒1.882; 1.082)
CJPET	TG	10.43 (5.91)	5.14 (4.26)	0.037 (0.429; 10.142) *
	CG	9.00 (8.00)	10.40 (6.99)	0.263 (‒4.390; 1.590)

Abbreviations: TG: Treatment Group; CG: Control Group; CI: confidence interval; SD: Standard Deviation; AROM: Active Range of Motion; CJPET: Cervical Joint Position Error Test; * = Significant comparison (*p* < 0.05).
